# Harnessing conversational generative artificial intelligence and natural language processing to address the colorectal cancer screening adherence gap: the Azores endeavour

**DOI:** 10.3389/fpubh.2026.1968297

**Published:** 2026-09-30

**Authors:** Francisco Goiana-da-Silva, Duarte Tude-Graça, Filipa Patrícia Gonçalves Correia, Rik Renard, João Pereira, Tomás Portas, João Perestrelo Borba, Hutan Ashrafian, João Carlos Cruz Barbosa de Macedo

**Affiliations:** 1 Centre for Health Policy, Institute of Global Health Innovation, Imperial College London, London, United Kingdom; 2 NOVA Medical School, Universidade NOVA de Lisboa, Lisbon, Portugal; 3 Faculdade de Ciências da Saúde, Universidade da Beira Interior, Covilhã, Portugal; 4 Sword Health, Inc, Draper, UT, United States; 5 Faculdade de Medicina, Universidade de Lisboa, Lisbon, Portugal; 6 Centre for Public Administration and Public Policies, Institute of Social and Political Sciences, University of Lisbon, Lisbon, Portugal; 7 Azores Oncology Centre, Angra do Heroísmo, Azores, Portugal; 8 Department of Surgery and Cancer, Faculty of Medicine, Imperial College London, London, United Kingdom

**Keywords:** adherence, ageing population, cancer screening, colorectal cancer, conversational AI, digital health, large language models, natural language processing

## Abstract

Organised colorectal cancer (CRC) screening reduces mortality, yet participation routinely falls short of policy targets, a gap most acute in structurally disadvantaged, insular, and rapidly ageing populations. The Azores, an outermost European Union archipelago, exemplifies this challenge: CRC accounts for 10% of regional cancer diagnoses and is the second leading oncological cause of potential years of life lost, with 2024 mortality (24.9 per 100,000) exceeding mainland Portugal. Despite a mature organised screening programme (ROCCRA), archipelago-wide participation stands at just 30.3%, with roughly one in five residents who consent and receive a home test kit never returning it. This perspective article argues that conversational artificial intelligence based on large language models (LLMs), the current generation of natural language processing (NLP) technology, capable of understanding and responding to patient concerns in real time, represents a promising approach for bridging this gap. Because the barriers identified in the literature are well, inherently dialogic, an interactive, multi-channel conversational cascade is better suited to address cognitive and operational friction than static reminders. Specifically, this paper synthesises presumed drivers of non-adherence, inferred from the literature and considered likely to apply to the Azorean context, reviews the evidence supporting conversational and voice-enabled outreach in cancer screening, and uses regional data to model the potential impact of a well-targeted voice AI deployment. Beyond the Azores, this framework illustrates how NLP-enabled conversational AI could support more responsive, equitable, and scalable public health screening programmes in geographically dispersed and underserved populations.

## Introduction

Colorectal cancer (CRC) is amongst the most common cancers for which population-level screening improves prognosis given that detecting precancerous lesions or diagnosing tumours at an earlier stage significantly reduces mortality (1). Nationally, colon and rectal cancers combined constitute one of the most incident cancer types in Portugal, accounting for over 7,800 new diagnoses annually, 4,591 in men and 3,230 in women, with incidence consistently higher in males across age groups (2). Whilst organised, invitation-based programmes utilising home-based Faecal Immunochemical Tests (FIT) are the gold standard for early detection, their success relies entirely on high population adherence. Despite decades of evidence supporting these programmes, participation rates across Europe remain highly heterogeneous ranging from roughly 12 to 68%, and frequently fall below the thresholds necessary for population-level impact (3, 4).

Whilst structural barriers, such as geographic isolation, rapid demographic ageing (5), and variable access to primary care (6, 7), partially explain this shortfall, a substantial proportion of non-adherence arises from behavioural and cognitive factors. In home-based FIT programmes, target individuals frequently grapple with low health literacy, affective aversion to stool sampling, procrastination, and self-efficacy anxiety regarding the unguided self-collection process. Overcoming these behavioural barriers requires more than resolving logistical bottlenecks, it demands targeted communication.

Recent advances in conversational artificial intelligence powered by natural language processing (NLP) have created new opportunities to address these barriers (8, 9). Unlike static reminders or conventional automated telephone systems, or earlier rule-based NLP agents constrained to predefined intents, LLM-based conversational agents can interpret patient questions expressed in open natural language, provide context-specific responses, reinforce behavioural motivation, and offer real-time procedural guidance whilst maintaining a consistent, auditable clinical knowledge base (8, 10). These capabilities position conversational AI as a valuable strategy for organised population screening programmes (9, 10).

The Azores, an autonomous region of Portugal and one of the European Union’s outermost regions, offer a clear illustration of this paradigm. The archipelago faces a substantial and worsening CRC burden. In 2024, the region recorded a standardised CRC mortality rate of 24.9 per 100,000 inhabitants, exceeding mainland Portugal (11). This mortality profile builds upon a sustained epidemiological signal: between 1997 and 2016, the region registered a standardised incidence rate of 36.5 per 100,000 inhabitants, with CRC accounting for 10.0% of all malignant neoplasms diagnosed locally (an average of 95.9 new cases annually) (12).

Despite completing at least three invitation cycles, the region’s organised CRC screening programme (ROCCRA) has plateaued below policy targets, with overall archipelago-wide participation standing at just 30.3%, marked by pronounced inter-island disparities ranging from 16.3% in Graciosa to 54.8% in Santa Maria.

This combination of an escalating cancer signal, documented structural disadvantage, and a mature screening programme struggling with localised friction provides both an urgent health concern and an invaluable research opportunity. The authors argue that this opportunity is best pursued through multi-channel conversational artificial intelligence, enabling adaptive dialogue capable of identifying individual barriers, responding to questions in real time, and tailoring behavioural support throughout the screening pathway. This case is reinforced by a broader European policy shift: Europe’s Beating Cancer Plan has made early detection one of its four pillars, and the European Commission’s Apply AI Strategy is now funding initiatives like the EUCanScreen Joint Action specifically to accelerate the adoption of artificial intelligence within cancer screening programmes (13). At the regional level, this strategic direction aligns with the Regional Programme for Combating Oncological Diseases. The programme establishes a 50% target for ROCCRA alongside a strategic action focused on using digitalization and artificial intelligence tools to complement procedural efficiency, increase screening participation rates, and enhance early diagnosis capabilities. This evolving policy landscape also underscores the growing importance of robust governance frameworks capable of ensuring transparency, accountability, clinical safety, and equitable deployment of AI-enabled public health interventions.

## Epidemiological and structural context

Within the Autonomous Region of the Azores, organised CRC screening targets an eligible population of approximately 76,927 adults aged 50–74 years. Longitudinal surveillance from the Azores Cancer Registry (ROA) reveals a concerning epidemiological divergence: whilst regional incidence and mortality for several major cancers have improved over time, CRC outcomes amongst men have moved in the opposite direction. Between 1997 and 2016, male standardised CRC incidence increased steadily, reaching 54.9 per 100,000 inhabitants during the 2012–2016 period and accounting for 11.0% of all male cancer diagnoses. Furthermore, in 2024, the male standardised CRC mortality reached 27.6 per 100,000 inhabitants, compared with 22.3 per 100,000 amongst women, reinforcing the need for targeted public health action to address this growing disease burden (14).

Against this background, the ROCCRA has generated longitudinal data across four invitation cycles. Despite the maturity of the programme, participation has remained remarkably stable at roughly 30%: 30.2% in Cycle 1, 29.7% in Cycle 2, 30.3% in Cycle 3, and 30.0% in Cycle 4 (9). Rather than demonstrating progressive improvement, these findings indicate that participation has plateaued well below regional policy ambitions.

As detailed in Table 1, the Cycle 3 archipelago average of 30.3% masks a more than three-fold variation across islands, ranging from a high of 54.8% in Santa Maria and 46.8% in Faial to a low of 16.3% in Graciosa and 20.9% in Flores. Crucially, the regional average is largely driven by São Miguel and Terceira, which together account for more than 78% of the eligible screening population (60,303 of 76,927 residents) and where uptake remains stagnant at 29.6 and 25.4%, respectively. Similar variability is evident within individual islands. In São Jorge, for example, screening uptake ranges from 34.3% in Calheta Primary Care Centre to 22.5% in Velas Primary Care Centre.

**Table 1:** CRC screening participation (Cycle 3) and primary care GP coverage across the Azores archipelago.

Health unit/Island	Target population (*N*)	Screened population (*n*)	Screening participation rate (%)	Family doctor coverage (%)
USI Corvo (Corvo)	110	44	40.0%	100.00%
USI Faial (CS Horta)	4,669	2,185	46.8%	70.73%
USI Flores (CS Sta. Cruz)	1,166	244	20.9%	98.86%
USI Graciosa (CS Sta. Cruz)	1,340	219	16.3%	69.22%
USI Pico	4,703	1,772	37.7%	71.70%
CS Lajes do Pico	1,427	586	41.1%	70.75%
CS Madalena	2,002	735	36.7%	74.10%
CS São Roque	1,274	451	35.4%	69.03%
USI Santa Maria (CS Vila do Porto)	1,681	921	54.8%	99.78%
USI São Jorge	2,955	809	27.4%	84.76%
CS Calheta	1,217	418	34.3%	99.47%
CS Velas	1,738	391	22.5%	74.38%
USI São Miguel	42,282	12,516	29.6%	95.53%
CS Nordeste	1,500	546	36.4%	99.61%
CS Ponta Delgada	27,511	7,857	28.6%	95.79%
CS Povoação	1,941	731	37.7%	99.83%
CS RIbeira Grande	7,968	2,356	29.6%	92.02%
CS Vila Franca do Campo	3,362	1,026	30.5%	94.02%
USI Terceira	18,021	4,577	25.4%	92.97%
CS Angra do Heroísmo	11,228	2,954	26.3%	91.56%
CS Praia da Vitória	6,793	1,623	23.9%	95.36%
Total Azores	76,927	23,287	30.3%	91.47%

To explore whether these disparities primarily reflect access to Primary Care, the authors examined the relationship between family doctor coverage and screening participation. This exploratory analysis identified a weak, statistically non-significant correlation between family doctor coverage and screening participation (*r* = 0.18; *p* = 0.25). This finding indicates only that the available ecological data did not demonstrate a statistically significant linear association between the two variables, it does not permit the conclusion that Primary Care capacity is not an important determinant of participation. Because Table 1 combines island-level and health-centre-level units, this analysis should be regarded as descriptive and hypothesis-generating rather than a formal epidemiological test of the determinants of participation. The observed variation across islands and health centres may instead reflect complex interactions between local implementation factors, island-specific logistical constraints, and behavioural determinants that were not directly assessed here.

## Where adherence is lost within the screening pathway

Designing effective implementation strategies requires disaggregating overall non-participation into stage-specific attrition along the screening continuum, as distinct behavioural and structural mechanisms govern drop-off at each phase (3, 15). In screening programmes that require affirmative consent prior to kit dispatch, like the Azores opt-in system (as shown in Figure 1), two primary drop-off points emerge: failure to respond to the initial invitation and failure to complete and return the FIT kit after consent has been provided.

**Figure 1:**
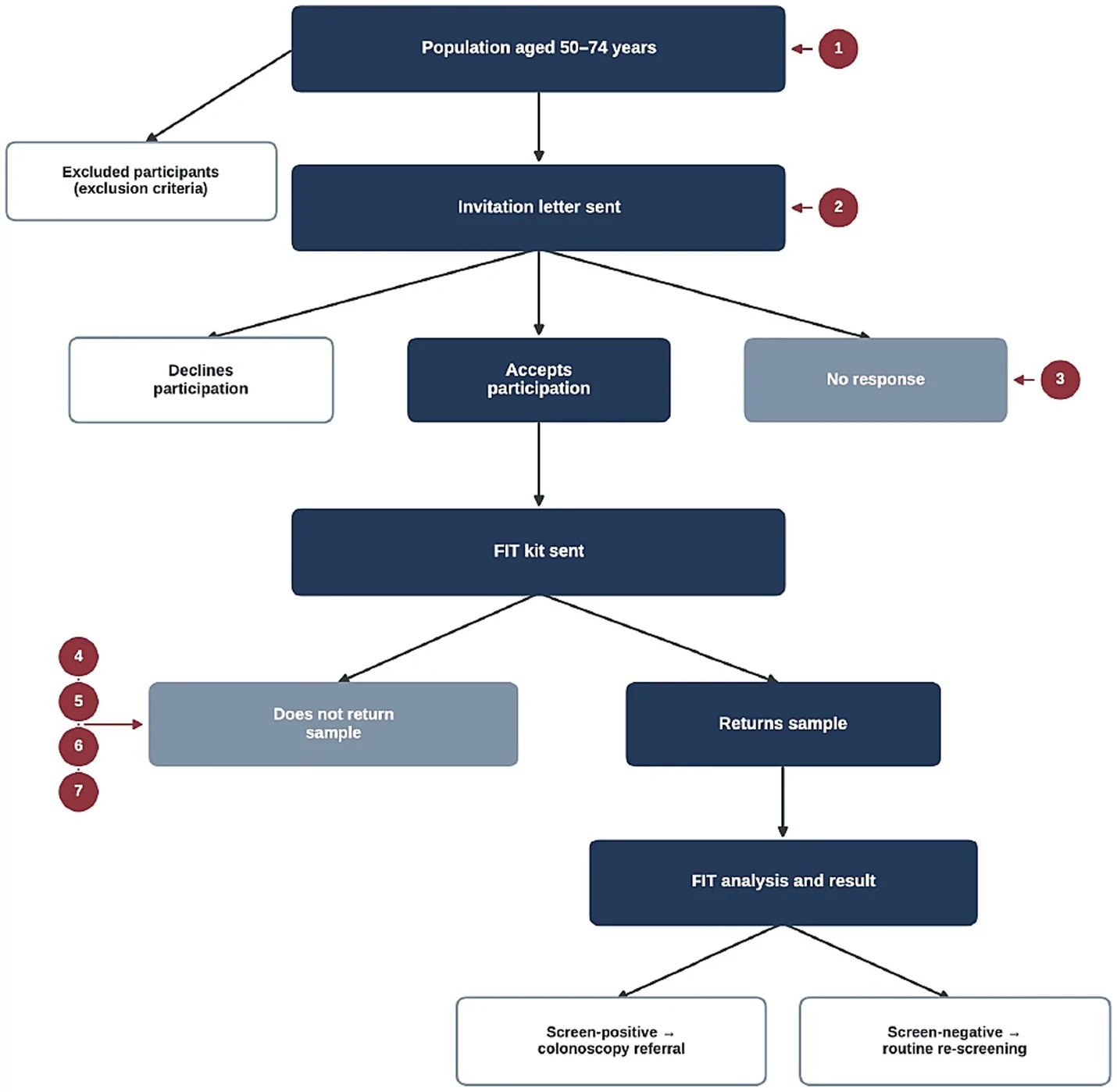
Organised CRC screening pathway with evidence-based intervention points. The figure illustrates the proposed organised CRC screening pathway and highlights seven evidence-based intervention points designed to improve participation and optimise programme performance. The numbered intervention points correspond to published strategies evaluated in previous studies. A detailed description of the supporting evidence for each intervention is provided in Supplementary Table S1 (16–22).

Because complete stage-specific pathway data are currently available only for the third screening cycle, these data were used to characterise attrition across the screening pathway. This data reveals a marked disconnect between initial willingness to participate and successful completion of screening. Although 37.9% of eligible invited residents provided informed consent to participate, overall programme participation reached only 30.3%. Consequently, approximately one in five consenting individuals (20.1%) who accepted a FIT kit ultimately failed to return a sample for laboratory analysis.

This post-consent attrition is consistent with the well-described intention–behaviour gap, whereby expressed willingness to participate does not necessarily translate into completion of the intended health behaviour, and therefore represents a fundamentally different implementation challenge from initial non-response (23). Individuals who do not respond to the initial invitation are more likely to be influenced by low perceived susceptibility, limited awareness of screening benefits, competing priorities, and other psychosocial barriers to participation (24–26). In contrast, post-consent non-returners have already expressed an explicit intention to participate. Their failure to complete screening is therefore more likely to reflect modifiable cognitive, emotional, and operational barriers arising during home-based self-testing, including self-efficacy concerns regarding sample collection, affective aversion to stool handling, procrastination, forgetfulness, or practical difficulties returning the kit.

Distinguishing between these two stages of attrition is critical because each requires fundamentally different implementation strategies.

## Individual, psychological, and operational drivers of non-adherence

The drivers below are drawn from the international CRC screening literature, none has yet been directly confirmed in the Azorean population, and this gap is itself part of the case for a conversational, rather than static, channel. Instead of assuming which barriers are present, LLM-based conversational AI has the potential to identify, through real-time dialogue, the specific cognitive, emotional, or operational barriers affecting each individual and to tailor its responses accordingly.

These two drop-off points may involve a complex interplay of individual cognitive perceptions, emotional responses, and operational challenges inherent to stool-based screening (24, 27).

Primary non-response, the main bottleneck holding consent at 37.9%, is hypothesised to reflect low perceived susceptibility and cancer fatalism. Because early-stage colorectal lesions are clinically silent, asymptomatic individuals frequently equate the absence of symptoms with good health, concluding that screening is unnecessary (25, 27). Furthermore, cancer fatalism triggers psychological defence mechanisms like denial and active avoidance. Expanding initial consent requires conversational engagement that proactively corrects risk misperceptions and dismantles fatalistic beliefs (28).

Conversely, post-consent attrition is plausibly linked to affective aversion, self-efficacy anxiety, and temporal discounting. Stool sampling elicits visceral feelings of discomfort and disgust (24). On an operational level, unguided home self-testing introduces substantial friction: individuals experience self-efficacy anxiety, fearing they lack the competence to collect the sample (6, 26). Finally, without rigid deadlines, execution is uniquely vulnerable to present bias, cognitive procrastination, and memory decay (26).

Consequently, improving participation towards the regional target of 50% is likely to require a dual-action conversational strategy that both increases initial consent through personalised behavioural engagement and supports successful FIT completion through real-time procedural guidance and reassurance.

## Emerging opportunities for personalised and technology-enabled outreach

The default strategy for addressing non-adherence in most organised screening programmes has traditionally relied on follow-up letters or short message service (SMS) reminders. A systematic review across breast, cervical, and CRC screening found that whilst SMS reminders achieved absolute participation gains of 4.5–15.0% in breast and cervical programmes, they produced a marginal increase of only 0.6–3.3% in CRC screening (29). These findings suggest that static, unidirectional communication is insufficient to overcome the behavioural, emotional, and procedural barriers.

A closer examination of reminder trials suggests that intervention efficacy depends less on the delivery medium itself and more on stage-specific matching. Two landmark trials within Catalonia’s FIT-based screening programme illustrate this principle. When SMS reminders targeted individuals who had already requested and received a FIT kit but failed to return it within 14 days, kit completion increased significantly compared with usual care (64.2% versus 53.7%) (30). Conversely, when a separate trial replaced introductory invitation letters with blanket SMS outreach to all initial non-responders, the intervention failed to improve participation and was terminated early (31).

Together, these findings highlight an important implementation principle. Static reminders are capable of prompting action when motivation already exists, but they are considerably less effective when individuals remain disengaged.

Advances within NLP, specifically the shift from rule-based and intent-classification systems to generative large language models, offer a fundamentally different implementation approach. Earlier conversational systems could only recognise a predefined set of intents; generative models can interpret patient intent from unconstrained natural language, respond dynamically to unanticipated questions, and personalise behavioural support in real time, whilst maintaining clinically consistent responses by grounding outputs in validated, auditable knowledge sources (32, 33). Notably, no cited study has yet evaluated a generative LLM-based Voice AI system directly against invitation response or FIT return. Voice AI represents one practical interface through which these capabilities can be delivered, combining the scalability of automated outreach with the adaptive support traditionally associated with clinician-led telephone navigation (34). Rather than replacing existing reminder systems, conversational AI has the potential to augment them by transforming communication from a one-way notification process into an interactive dialogue. It should be noted that the evidence reviewed above spans distinct technologies and outcomes: interactive voice-response trials that predate generative AI, a randomised trial of AI-generated messaging on screening intent, a chatbot protocol not yet reporting completed-screening outcomes, and a narrative account of the anticipated capabilities of generative voice agents.

Figure 1 proposes an evidence-informed implementation pathway. The supporting evidence underpinning each intervention point is summarised in Supplementary Table S1 (16–22).

## Voice AI as an emerging solution for the Azores

Addressing the persistent participation gap in CRC screening requires moving beyond traditional, unidirectional reminders towards an interactive, multi-channel implementation strategy capable of adapting communication to the behavioural needs of individual participants. Whilst digital messaging platforms, such as SMS and WhatsApp, provide broad population reach at low marginal cost, static notifications rarely overcome the complex behavioural barriers that characterise home-based FIT screening (6). Conversely, conversational interactions allow concerns to be explored and addressed in real time, providing the reassurance, procedural guidance, and behavioural support required to sustain participation throughout the screening pathway. This logic mirrors the stepped-care principle already validated in CRC screening, escalating the intensity of contact only for those who do not respond to lower-intensity outreach (35). Within this framework, behaviourally designed digital messaging serves as the initial engagement strategy, whilst LLM-based conversational AI is proposed as the escalation layer for individuals who remain disengaged or experience difficulties completing the screening process. Evaluation of the Azores’ ROCCRA programme demonstrates why such a dual-stage implementation strategy is necessary.

This sequential structure has important implications for intervention design. Even if an intervention were capable of eliminating post-consent attrition entirely by achieving universal kit return amongst consenting individuals, overall programme participation could not exceed the initial consent rate of 37.9%. Improving kit completion alone is therefore mathematically insufficient to achieve the regional policy objective of 50% participation. Meaningful improvement requires simultaneous action at both stages of the pathway: increasing the proportion of eligible individuals who consent to screening whilst reducing attrition amongst those who have already entered the programme.

The following calculations are illustrative scenario analyses built from external, evidence-informed effect sizes, they are not estimates of the effect of Voice AI, whose real-world effectiveness has not been tested, and should not be read as predictions of programme performance. To illustrate the potential population impact of such an approach, published intervention effect estimates were applied to the ROCCRA baseline, subject to the caveats set out below.

At the invitation stage, a randomised trial conducted within the Catalan organised screening programme demonstrated that a brief conversational telephone call delivered before the standard invitation letter increased participation by 10.7 percentage points (36). Applied to the Azorean baseline, an effect of similar magnitude would increase initial consent from 37.9% to approximately 48.6%. At the post-consent stage, the M-TICS trial demonstrated a 10.5-percentage-point improvement in FIT return amongst individuals who had received a kit but had not returned it (30). Applying a comparable effect to the ROCCRA population would increase kit return from 79.9% to approximately 90.4%. Assuming both effects were achieved simultaneously, overall participation would increase from 30.3% to approximately 43.9%, corresponding to an estimated 10,510 additional completed screening tests within a single invitation cycle. A more conservative scenario, assuming only half of the published effect size at each stage, would still increase participation to approximately 36.8%, representing around 5,040 additional completed FIT tests. These estimates should be interpreted as illustrative implementation scenarios rather than predictions of programme performance. The 10.7-percentage-point and 10.5-percentage-point effects derive, respectively, from a live telephone intervention delivered within a different outreach programme and from SMS reminders sent to a subgroup of participants who had already received a FIT kit but had not returned it. Both differ in modality, target population, and denominator from a generative, LLM-based Voice AI intervention applied to the full Azorean screening population. The effects of human-delivered calls or text messages should therefore not be assumed to transfer directly to LLM-based conversational AI, and the figures above are presented strictly as external, evidence-informed scenarios rather than anticipated effects of the proposed intervention.

Nevertheless, they demonstrate that achieving the regional policy target of 50% will probably require not only improvements greater than those currently reported in the literature but also sustained implementation across successive screening rounds and the integration of complementary interventions alongside conversational AI.

Although these projections are presented from an implementation perspective, their implications are ultimately clinical. Evidence from FIT-based screening programmes indicates CRC detection rates of approximately 0.3–0.7% and advanced adenoma detection rates of 1.3–2.1% amongst completed tests (37, 38). Applied to the conservative implementation scenario described above, and assuming an equivalent diagnostic yield amongst the additional participants, this corresponds to an estimated 14–35 additional CRC diagnoses and 64–106 advanced adenomas that could potentially be identified. The detection-rate data used do not indicate the tumour stage of these additional cases, and no inference about stage at diagnosis can be drawn from this calculation.

Even modest improvements in screening participation therefore have the potential to translate into clinically meaningful gains at the population level.

Beyond clinical and diagnostic gains, the operational and health-economic impact of conversational AI represents a critical dimension for prospective evaluation. Currently, manual telephone outreach to persuade non-participants and explore reasons for non-response relies heavily on dedicated personnel with substantial time, administrative, and financial costs. Leveraging conversational Voice AI to automate these interactions has the potential to optimise resource allocation, liberating valuable human workforce hours to be redirected towards direct clinical care and complex patient management.

The implementation of conversational AI must be accompanied by robust governance frameworks. Individuals should always be informed when interacting with an AI system, and all clinical content should be derived from validated, auditable knowledge sources capable of ensuring consistency with established screening recommendations. Clear boundaries must distinguish procedural guidance from personalised medical advice, with predefined escalation pathways for situations requiring clinician involvement. More broadly, future implementation should be accompanied by prospective evaluation of safety, effectiveness, equity, transparency, and human oversight to ensure that AI-enabled conversational systems strengthen, rather than replace, existing organised screening programmes.

## Conclusion

Improving participation in organised CRC screening requires implementation strategies that move beyond uniform, unidirectional communication. The findings presented in this perspective suggest that conversational artificial intelligence powered by NLP offers a promising implementation approach by enabling personalised, real-time behavioural support whilst maintaining clinically consistent and auditable interactions.

Using the Azores as a real-world implementation case, this perspective highlights how epidemiological need, geographical dispersion, and persistent participation gaps converge to create a unique setting for evaluating conversational AI within an organised population screening programme. Modelling based on regional screening data suggests that a dual-stage implementation strategy addressing both initial participation and post-consent attrition could achieve clinically meaningful improvements in screening uptake. Even conservative implementation scenarios could lead to thousands of additional completed FIT tests and substantially earlier detection of CRC and advanced adenomas at the population level.

The projections presented in this perspective are inherently theoretical and now require rigorous prospective evaluation under real-world conditions. Demonstrating these anticipated benefits within the Azorean CRC screening programme will be essential to establish the evidence base for this implementation strategy. If confirmed through prospective real-world evaluation, the Azorean experience could inform implementation and policy decisions in other Portuguese regions whilst providing transferable evidence for organised population screening programmes internationally.

Although centred on CRC screening in the Azores, the implementation framework proposed here has broader relevance. More generally, it illustrates how NLP-enabled conversational AI can support more responsive, equitable, and scalable public health programmes. As health systems increasingly seek to expand preventive care whilst facing workforce constraints and growing service demand, conversational AI represents not simply a new communication technology, but a promising implementation strategy for strengthening organised population health programmes.

## Data Availability

The original contributions presented in the study are included in the article/Supplementary material, further inquiries can be directed to the corresponding author/s.
