# Supplementary material for: Harnessing conversational generative artificial intelligence and natural language processing to address the colorectal cancer screening adherence gap: the Azores endeavour
Source: Front Public Health. 2026 Sep 30;14:1968297. doi: 10.3389/fpubh.2026.1968297 (PMC13662140; doi:10.3389/fpubh.2026.1968297)
Supplement: Supplementary file 1 [file Table_1.docx]

| **Intervention Point** | **Screening stage** | **Evidence-based intervention** | **Main reported outcome** | **References** |
| --- | --- | --- | --- | --- |
| 1 | Pre-invitation | Risk-stratified invitation using a validated CRC risk score combined with personalised screening recommendations and follow-up. | Screening uptake increased from 33.3% to 63.3% compared with usual care. | Fok et al., 2025 ^(32)^ |
| 2 | Invitation | Direct mailing of FIT kits, with optional telephone assistance or nurse navigation. | Direct FIT mailing increased adherence by 24.5%, with additional improvements from telephone support and nurse navigation. | Green et al., 2022^(33)^ |
| 3 | Eight weeks after invitation | Reminder strategy including behavioural letter and/or direct FIT mailing for non-responders. | Direct FIT mailing significantly increased screening completion; behavioural letters alone showed limited benefit. | Clarke et al., 2025 ^(34)^ |
| 4 | FIT not returned | Sequential stepped-care reminder pathway using letters, automated calls or text messages, followed by live telephone navigation. | Progressive increases in FIT return were observed throughout the escalation pathway, with participant engagement predicting higher completion rates. | Baker et al., 2014 ^(35)^ |
| 5 | FIT not returned | Automated educational SMS programme with repeated reminders until FIT return or opt-out. | Feasible digital reminder strategy; comparative effectiveness was not reported in the original study. | Kim et al., 2020 ^(36)^ |
| 6 | FIT not returned | Multimodal reminder programme comparing letters, automated calls, SMS messages and live telephone calls. | Reminder interventions produced an additional 25.5% FIT return, with personalised telephone calls achieving the greatest effect. | Coronado et al., 2017 ^(37)^ |
| 7 | FIT not returned | Behavioural planning tool combined with predefined return deadlines. | A suggested two-week deadline produced the greatest reduction in reminder requirements. | Robb et al., 2025 ^(38)^ |
